# A contribution to the French validation of the clinical anxiety scale amongst health care workers in Switzerland

**DOI:** 10.1186/s40359-024-01525-y

**Published:** 2024-01-19

**Authors:** Yana Apostolova, Elisabeth Stamm, Francesco Cilla, Anne-Véronique Durst, Christophe Büla, Patrizia D’Amelio

**Affiliations:** 1 Service of Geriatric Medicine and Geriatric Rehabilitation, Lausanne University Hospital and University of Lausanne, Route de Mont Paisible 16, 1011 Lausanne, Switzerland; 2 Service of Geriatric Medicine, HFR Freiburg Kantonsspital, Freiburg, Switzerland

**Keywords:** Clinical anxiety scale, CAS, French, Psychometric properties

## Abstract

**Background:**

Anxiety disorders are frequent but remain often underdiagnosed and undertreated. Hence, valid screening instruments are needed to enhance the diagnostic process. The Clinical Anxiety Scale (CAS) is a 25-item anxiety screening tool derived from the Hamilton Anxiety Scale (HAM-A). However, this scale is not available in French. The General anxiety disorder − 7 (GAD-7) scale, which has been validated in French, is a 7-item instrument with good psychometric properties. This study contributes to the validation of an adapted French version of the CAS, using the GAD-7 as the reference.

**Methods:**

A forward-backward English-French-English translation of the CAS was performed according to standard practice. The French versions of the CAS and GAD-7 were completed by 127 French speaking healthcare professionals. CAS internal consistency was assessed using Crohnbach’s alpha, and test-retest reliability was tested after 15 days in a subsample of 30 subjects. Convergent validity with GAD-7 was assessed using Pearson’s correlation coefficient. Test-retest reliability was explored using one-way random effects model to calculate the intra-class correlation coefficient (ICC).

**Results:**

French CAS showed excellent internal consistency (Cronbach’s alpha 0.97), high convergent validity with GAD-7 (Pearson’s R 0.81, *p* < 0.001), and very good test-retest reliability (ICC = 0.97, 95% CI 0.93–0.98).

**Conclusion:**

The proposed French version of the CAS showed high reliability and validity that need to be further investigated in different populations.

## Introduction

Anxiety disorders i.e., experiencing symptoms of excessive fear and worry that result in behavioural disturbances, are leading mental health problems [1]. According to a recent epidemiological survey by Yang et al. [2] the number of persons newly diagnosed with anxiety disorders has increased over the last 30 years. Moreover, the burden of anxiety and major depressive disorders raised further during the COVID-19 pandemic [3].

Anxiety disorders are associated with adverse health outcomes, contribute to poor quality of life and increased mortality [4]. According to the Global Burden of Diseases 2019 Study, anxiety disorders are amongst the leading causes of disability, responsible for about 28.7 million disability-adjusted life years [5]. In Switzerland we are witnessing a rise in the incidence of psychological distress, and 11.9% of the women ant 7.5% in 2022, suffer from anxiety disorder [38].

Early diagnosis and intervention may reduce the disease burden and improve the quality of life of patients affected by anxiety [6]. Yet, despite the high prevalence and substantial disability associated with these disorders, they often remain underdiagnosed and undertreated [7–9]. Hence, there is a need for valid and accurate screening instruments to enhance the diagnostic process.

Several screening instruments have been developed to effectively identify patients with anxiety disorders, however only few are adapted for local languages and culture. The broadly used scales for anxiety (such as HAD or GAD7) have been validates in French population [39–40], but none in the French speaking Switzerland.

One of the most widely used scale is the General Anxiety Disorder-7 (GAD-7), a seven-item instrument with good psychometric properties [42] (Cronbach’s alpha 0.92, test-retest reliability, intra-class correlation 0.83). The convergent validity of the GAD-7, was demonstrated by its correlations with two anxiety scales: the Beck Anxiety Inventory (*r* = 0.72) and the anxiety subscale of the Symptom Checklist-90 (*r* = 0.74) [13].

Since its development by Spitzer et al., GAD-7 has been validated in different populations such as psychiatric patients, patients from primary care clinics, patients affected by specific diseases or health problems such as epilepsy, heart failure, or traumatic brain injury, as well as in the general population [14–20]. GAD-7 is translated in more than 50 languages. The scale is short and easy to complete. The GAD-7 targets mainly the General Anxiety disorder and does not include specific features of other types of anxiety disorders such as panic, phobias, and post-traumatic stress disorders. Here, we selected the GAD-7 as our standard reference due to its high specificity and sensitivity for detecting anxiety [22]. This choice reduces the risk of potential bias stemming from the inclusion of psychotic symptoms or depression, as demonstrated in previous research with other scales as the SCL-90R [36]. Therefore, the GAD-7 is well-suited for identifying anxiety within a general population and aligns with the objectives of our study.

The Clinical Anxiety Scale (CAS) is a 25-item tool derived from the Hamilton Anxiety Scale (HAM-A). HAM-A is still used in clinical practice and in research and comprises items covering a wide range of anxiety including multiple somatic symptoms as well as some depressive symptoms. HAM-A contains 14 items, each scored on a scale of 0 (not present) to 4 (severe). This scoring system results in a total score range of 0 to 56, which reflects the varying intensity of anxiety symptoms However, some concerns have been raised on its inaccuracy in discriminating somatic anxiety from antidepressant side effects [10], and for its time-consuming and potentially unreliable administration by physicians [11, 27].

CAS is simpler in comparison to HAM-A. CAS comprises predefined questions without subscales, making it straightforward for self-administration, unlike HAM-A which need to be administered by a health care professional. It targets essentially anxiety symptoms, thus excluding the potential bias of questions related to depression. CAS assesses the level of anxiety arising from identified situations or events. Compared to GAD-7, CAS is longer and combines a wider range of questions concerning panic and phobia, and few somatic symptoms of anxiety [12]. Thus, CAS could be especially useful to detect specific types of anxiety rather than general anxiety disorder.

The psychometric properties of CAS established in the original validation article [12] are very good. CAS achieved a Cronbach Alpha Coefficient of 0.94. Its discriminant validity of 0.77 was better, compared to other scales (Index of Family Relations, General Contentment Scale, Psycho-Social Screening Package, Mobility Inventory Agoraphobia, and Michigan Alcoholism Screening Test). It is well correlated and has a good concurrent validity with the anxiety subscale (HAD-A) of the HAD (Hospital Anxiety Depression scale) (correlation coefficients 0.69–075), [31–33] and good temporal stability [34].

Despite these positive characteristics, CAS is only available in English. Furthermore, studies examining the factorial structure of this scale are lacking.

The aim of this study was to develop and validate a French version of the CAS, to examine the internal consistency, the factorial structure with principal component analysis, as well as to assess the construct validity using GAD-7 as a reference and evaluate its test-retest reliability.

## Methods

### Translation of the scale

Two bilingual experts performed a forward-backward English-French-English translation of the CAS. The two translated forms displayed very good similarity. The final version was reviewed by a bilingual psychologist and subsequently used in the study.

### Procedure and participants

The CAS includes 25 items, with answers ranging from 1 (rarely) to 5 (very often). After reverse scoring of the positively formulated items, the final scores range from 0 to 100 with higher scores indicating higher anxiety. The final score was calculated according to the formula provided in the original CAS validation paper [21]. A cut-off of 30 or more defines clinically significant anxiety.” [12, 21].

The GAD-7 comprises 7 items and the global score ranges from 0 to 21, with higher scores indicating higher anxiety. A score of 8 or more is usually proposed to define clinically significant anxiety [22].

Although 142 subjects were eligible for the study, only 127 subjects were recruited as 15 refused to participate. All participants were health care professionals working in the Lausanne University Hospital (CHUV) in different divisions: geriatrics, internal medicine, and psychiatry. The inclusion criteria were age 18 years or older, native French speakers or fluent in French, agreeing to participate. The participants were recruited between 21st September 2021 and 02nd February 2022.

Data on participants’ age, gender, and professional role was collected. All the participants completed the two self-administrated scales (CAS and GAD-7), using individual paper questionnaires. The two scales, completed in random order [35], had an identical response rate.

CAS test-retest reliability was examined in 30 participants (24%) who were asked to complete again both questionnaires 15 [43] days after their initial assessment, the sample size needed was calculated according to Walter et al. [36]. The time needed for the self-administration of the two questionnaires was measured subsequently in six subjects.

### Statistical analysis

The sample size was estimated according to Tabachnick et al. [23] - five subjects were needed to validate each item of the analysed scale, resulting in a sample size of 125 participants.

To evaluate the adequacy of the data for Factor Analysis, the Kaiser-Meyer-Olkin (KMO) Test and the Bartlett sphericity test were carried out. Subsequently, we carried out an exploratory principal components analysis with Varimax rotation with Kaiser normalization was performed on the responses to the 25 items of the CAS, to identify its factorial structure. Principal component and not factorial analyses was chosen because of the nature of the data obtained, the data did not exhibit clear underlying factors, and our goal was to capture as much variance as possible with a smaller number of variables. The Varimax rotation was chosen to avoid cross loadings on more than one dimension thus simplifying the factor structure and making each factor more interpretable in isolation. All CAS items were allowed to freely load during exploratory factor analysis to identify all factors present. Next, each factor loading was compared to determine the magnitude of difference. Differences in magnitude greater than 0.03 was set as the threshold for a stable factor structure [45].

The correlation between CAS and the GAD-7 was evaluated by Pearson’s correlation coefficient.

Test-retest reliability was assessed using a one-way random effects model to calculate the intraclass correlation coefficient (ICC), The intraclass correlation coefficient (ICC) is defined as a ratio of variability between subjects to the total variability including subject variability and error variability; as the error term decreases, the ICC moves from 0 to 1 indicating perfect reliability [24].

All the analyses were performed using SPSS 27.0 for Windows.

## Results

Overall, 127 of the 142 eligible health care professionals completed both questionnaires (response rate 89.4% for both instruments). Participants’ mean age was 35 ± 11 years, 61% were women, 45.6% were nurses, 22.0% physicians, 32.2% from other health professions (physical and occupational therapists, medical secretaries, medical and nurse students).

There was no significant difference in the CAS and GAD-7 scores between men and women as well as between the different professional categories (data not shown).

The mean time to complete the CAS and the GAD-7 were 120 s and 45 s, respectively.

The KMO (0.851) and the Bartlett sphericity tests (*p* < 0.001) indicated that the sample size was adequate and suitable for factor analyses.

The principal component factor analysis revealed seven principal components, however as there was only 1-item loading on the seventh factor (“I am free from senseless or unpleased thoughts”), the principal component analyses was carried out forcing the items on 6 loading according to Costello et al. [37].

This 6-factor structure of the CAS explained 66.83% of its total variance.

The ***first factor***, which explained 27.3% of the variance, encompassed the seven positively formulated questions related to “not worrying”. ***Factor 2*** which explain 17.12% of the variance had significant loadings on nine items related to Anxiety. ***Factor 3*** significantly loaded on eight items associated to panic and phobia that explained 8.31% of the variance. ***Factor 4*** included five items associated to Panic-Related Symptoms that explained 5.44% of the variance. Five items associated to Physical Symptoms loaded significantly on ***Factor 5*** that explained 4.5% of the variance, and finally only four items associated to Antidepressant and Tranquilizer Use on ***Factor 6*** that explained 4.2% of the total variance, respectively.

The result of our study supports a 6-factor structure of the CAS, each of which is associated with different components of anxiety. These factors can provide valuable insights into the multidimensional nature of anxiety, as follows: Factor 1: General Anxiety “I feel calm”; “I feel confident about the future”, “I feel relaxed and in control of myself”, “I feel generally anxious”. Factor 1 seems to be associated with general or non-specific feelings of anxiety. These items reflect a sense of overall anxiety or a lack of calmness and confidence about the future. This factor may capture a more generalized state of anxiety. Factor 2: Tension and Nervousness “I feel tense”; “I feel nervous”; “I feel nervousness or shakiness inside”. Factor 2 appears to be associated with feelings of tension and nervousness. These items reflect the psychological and physiological manifestations of anxiety. Factor 3: Fear and Avoidance: “I feel suddenly scared for no reason”; “I feel afraid to go out of my house alone”; “I feel afraid without good reason”; “Due to my fears, I unreasonably avoid certain animals, objects, or situations”. Factor 3 is associated with fear and avoidance behaviours. These items reflect unfounded fears and avoidance of various situations and objects, suggesting a specific type of anxiety related to phobias and avoidance behaviour.

Factor 4: Panic-Related Symptoms and Agoraphobia: “I have spells of terror or panic”; “I feel afraid in open spaces or in the streets”; “I feel afraid I will faint in public”; “I experience sudden attacks of panic which catch me by surprise”. Factor 4 appears to be related to panic-related symptoms and agoraphobia-like anxiety. These items represent experiences of sudden panic, fear in open spaces, and concerns about fainting in public. Factor 5: Physical Symptoms and Avoidance, “My hands, arms, or legs shake or tremble”; “I get upset easily or feel panicky unexpectedly”; “Due to my fears, I avoid being alone, whenever possible”. Factor 5 is associated with physical symptoms of anxiety, including trembling limbs and avoiding being alone due to fear. This factor may be related to social anxiety or specific phobias with physical symptoms. Factor 6: Medication Use, “I use tranquilizers or antidepressants to cope with my anxiety”; Factor 6 is primarily associated with the use of medication (tranquilizers or antidepressants) as a coping strategy for anxiety. This factor reflects a different aspect of managing anxiety.

The Varimax rotated component matrix (with Kaiser normalization) showed that most items had highest loading on the first (Not worrying: 0.80–0.91), second (Anxiety: 0.64–0.84) and third (Panic/Phobia: 0.57–0.83) factors (Table 1).

**Table 1:** Rotated Varimax factors structure of the French version of the CAS

	Factors*					
	1	2	3	4	5	6
I feel calm	0.882					
I feel tense		0.726				
I feel suddenly scared for no reason		0.719				
I feel nervous		0.788				
I use tranquilizers or antidepressants to cope with my anxiety					0.710	
I feel confident about the future	0.863					
I am free from senseless or unpleasant thoughts	0.346					0.540
I feel afraid to go out of my house alone						0.711
I feel relaxed and in control of myself	0.848					
I have spells of terror or panic		0.482	0.528		0.371	
I feel afraid in open spaces or in the streets			0.670			
I feel afraid I will faint in public			0.619	0.397	0.334	
I am comfortable traveling on buses, subways, or trains	0.888					
I feel nervousness or shakiness inside		0.708				
I feel comfortable in crowds, such as shopping or at a movie	0.808					
I feel comfortable when I am left alone	0.888					
I feel afraid without good reason		0.674				
Due to my fears, I unreasonably avoid certain animals, objects or situations			0.810			
I get upset easily or feel panicky unexpectedly		0.490	0.367	0.401		-0.306
My hands, arms or legs shake or tremble.			0.312	0.680		0.322
Due to my fears, I avoid social situations, whenever possible			0.712			
I experience sudden attacks of panic which catch me by surprise			0.363	0.448	0.333	
I feel generally anxious		0.855				
I am bothered by dizzy spells					0.622	
Due to my fears, I avoid being alone, whenever possible				0.753		

### Reliability and convergent validity

CAS internal consistency coefficient was 0.97 according to Cronbach’s alpha and test-retest reliability after 15 days showed very good [41] temporal stability (ICC = 0.966, 95% CI 0.93–0.98).

The CAS French version displayed a significant [44] convergent validity with GAD-7 both at baseline (Pearson *r* = 0.81, *p* < 0.001) and at 15-day follow-up (*r* = 0.86, *p* < 0.001).

## Discussion

The aim of our study was to develop and validate a French version of the CAS, to make it available for detection of anxiety in the French speaking population [25]. The results showed that this translated CAS version had high reliability and validity (Reliability coefficient value > 0.9, validity coefficient > 0.4).

An original contribution of the present study is to provide new insight on the factor structure of the CAS. Indeed, information on principal component analysis (PCA) is not available for the original English scale [21]. Contemporary studies using CAS are sparse and focused on the correlation with other anxiety scales.

The CAS, with its 6-factor structure, provides a comprehensive assessment of various components of anxiety, ranging from general anxiety and tension to specific fears, panic-related symptoms, and coping mechanisms. Understanding these factors can help clinicians and researchers better target and address the diverse aspects of anxiety in individuals.

Further analysis in different populations is needed to confirm the proposed this structure of the CAS and its French version.

The French CAS showed excellent reliability. Internal consistency was high, indicating it is highly homogeneous, as reported for the English version [21]. Similarly, test-retest reliability was also very high (*r* > 0.9) [41] the present study, emphasizing CAS stability over time in the absence of new events.

Further studies are welcome to investigate the sensitivity to changes of the French CAS amongst subjects developing new symptoms of anxiety.

Our results show an excellent convergent validity with the GAD-7 ( *r* = 0.81) thus confirming the link between the core symptoms of general anxiety disorders evaluated by the GAD-7 and the psychological and somatic symptoms of anxiety explored with the French CAS.

The initial validation of the scale was performed in a mixed population of subjects with and without a clinical diagnosis of anxiety or related disorders [21]. However, the present study differs as participants were health care workers, and the specific characteristics of this cohort, may limit the generalizability of our results, in particular the high homogeneity of the participant sample could potentially favor high correlation coefficients, thereby creating a limitation in the interpretation of the study results. Nevertheless, our results are consistent with other studies performed in the general population with different scales (HAD and STAI) [28–30]. Overall, 11.8% of the participants reached the cuff-off of CAS for anxiety, not dissimilar to those studies on the general population of the same age. Further research is needed to investigate the diagnostic validity of this instrument in subject affected by anxiety disorders.

Overall response rate for the questionnaire was high, however, the use of the scale in general population, not affected by anxiety, can also explain the high response rate.

A positive characteristic of the CAS is the self-administration and speed of completion that allow it to be used on a large scale in clinic. The present study is thus reassuring about the feasibility to use this CAS French version. In particular, the time needed to fill in the questionnaire was less than 3 min, confirming that this scale could likely be used in routine clinical practice by the general practitioner’s as well as in hospital setting, similarly to the English version. This is especially interesting when comparing to other, more time-consuming instruments such as the HAM-A [26].

## Conclusion

The validation of the French version of the CAS allows clinicians to assess anxiety disorders in a quick and efficient manner. The instrument is well accepted and could be included in routine clinical practice. Further studies are needed to clinically validate this scale in different populations, including in older patients.

## Data Availability

The datasets used and/or analysed during the current study are available from the corresponding author on reasonable request.

## Abbreviations

CAS: Clinical anxiety scale
GAD-7: General Anxiety Disorder- 7
ICC: Intra-class correlation coefficient
AD: anxiety disorders
HAM-A: Hamilton anxiety scale
HADS-A: Hospital Anxiety
PTSD: post-traumatic stress disorder
KMO: Kaiser-Meyer-Olkin
PCA: Principal component analysis
